# Health Care Providers and the Public Reporting of Nursing Home Quality in the United States Department of Veterans Affairs: Protocol for a Mixed Methods Pilot Study

**DOI:** 10.2196/23516

**Published:** 2021-07-21

**Authors:** Camilla B Pimentel, Valerie Clark, Amy W Baughman, Dan R Berlowitz, Heather Davila, Whitney L Mills, David C Mohr, Jennifer L Sullivan, Christine W Hartmann

**Affiliations:** 1 Center for Healthcare Organization and Implementation Research United States Department of Veterans Affairs Bedford Healthcare System Bedford, MA United States; 2 New England Geriatric Research Education and Clinical Center United States Department of Veterans Affairs Bedford Healthcare System Bedford, MA United States; 3 Division of Health Informatics and Implementation Science Department of Population and Quantitative Health Sciences University of Massachusetts Medical School Worcester, MA United States; 4 Department of Medicine Massachusetts General Hospital Boston, MA United States; 5 Department of Public Health Zuckerberg College of Health Sciences University of Massachusetts Lowell Lowell, MA United States; 6 Center for Healthcare Organization and Implementation Research United States Department of Veterans Affairs Boston Healthcare System Boston, MA United States; 7 Center of Innovation in Long Term Services and Supports United States Department of Veterans Affairs Providence Healthcare System Providence, RI United States; 8 Department of Health Services, Policy and Practice School of Public Health Brown University Providence, RI United States; 9 Department of Health Law, Policy and Management School of Public Health Boston University Boston, MA United States

**Keywords:** nursing homes, public reporting, quality

## Abstract

**Background:**

In June 2018, the United States Department of Veterans Affairs (VA) began the public reporting of its 134 Community Living Centers’ (CLCs) overall quality by using a 5-star rating system based on data from the national quality measures captured in CLC Compare. Given the private sector’s positive experience with report cards, this is a seminal moment for stimulating measurable quality improvements in CLCs. However, the public reporting of CLC Compare data raises substantial and immediate implications for CLCs. The report cards, for example, facilitate comparisons between CLCs and community nursing homes in which CLCs generally fare worse. This may lead to staff anxiety and potentially unintended consequences. Additionally, CLC Compare is designed to spur improvement, yet the motivating aspects of the report cards are unknown. Understanding staff attitudes and early responses is a critical first step in building the capacity for public reporting to spur quality.

**Objective:**

We will adapt an existing community nursing home public reporting survey to reveal important leverage points and support CLCs’ quality improvement efforts. Our work will be grounded in a conceptual framework of strategic orientation. We have 2 aims. First, we will qualitatively examine CLC staff reactions to CLC Compare. Second, we will adapt and expand upon an extant community nursing home survey to capture a broad range of responses and then pilot the adapted survey in CLCs.

**Methods:**

We will conduct interviews with staff at 3 CLCs (1 1-star CLC, 1 3-star CLC, and 1 5-star CLC) to identify staff actions taken in response to their CLCs’ public data; staff’s commitment to or difficulties with using CLC Compare; and factors that motivate staff to improve CLC quality. We will integrate these findings with our conceptual framework to adapt and expand a community nursing home survey to the current CLC environment. We will conduct cognitive interviews with staff in 1 CLC to refine survey items. We will then pilot the survey in 6 CLCs (2 1-star CLCs, 2 3-star CLCs, and 2 5-star CLCs) to assess the survey’s feasibility, acceptability, and preliminary psychometric properties.

**Results:**

We will develop a brief survey for use in a future national administration to identify system-wide responses to CLC Compare; evaluate the impact of CLC Compare on veterans’ clinical outcomes and satisfaction; and develop, test, and disseminate interventions to support the meaningful use of CLC Compare for quality improvement.

**Conclusions:**

The knowledge gained from this pilot study and from future work will help VA refine how CLC Compare is used, ensure that CLC staff understand and are motivated to use its quality data, and implement concrete actions to improve clinical quality. The products from this pilot study will also facilitate studies on the effects of public reporting in other critical VA clinical areas.

**International Registered Report Identifier (IRRID):**

DERR1-10.2196/23516

## Introduction

### Background

Public reporting seeks to improve quality by addressing informational asymmetries in health care [1]. This mechanism has been used in the nursing home sector since 2002, when the United States Centers for Medicare and Medicaid Services (CMS) launched the Nursing Home Compare website, which enables consumers to make choices based on quality [2,3]. These *report cards* theoretically incentivize nursing home providers to compete on quality by, for example, investing in quality improvement (QI) to maintain or increase market share [4]. Previous studies found that quality on some postacute quality measures improved after the launch of Nursing Home Compare, especially in nursing homes that began QI activities in response to their report cards [5] or that were subject to public reporting requirements [6]. Nursing home–level use of antipsychotic medications [7] and physical restraints [5], ambulation [6], and pain prevalence [4,5] improved, largely driven by actions that nursing home providers took to enhance care quality [4]. In a separate study, Mukamel et al [8] used the Nursing Home Compare survey to understand nursing home administrators’ responses to Nursing Home Compare. An initial survey in 2004 (724/1502, 48.2% response rate) found that although 80% of nursing home administrators had viewed their first report card, subsequent action depended on their perception of the validity of the scores [8]. Many administrators (40%) were ambivalent about the validity of quality measures. Nursing homes with poor scores were also more likely to act than nursing homes with better scores. Overall, 63% further investigated their scores, 42% changed the priorities of existing QI efforts, and 20% started new QI programs. A small but critical number of nursing homes used potentially dysfunctional strategies, that is, reallocating staff from other activities to care related to a poor-performing quality measure (a *teaching-to-the-test* response; 9%) and changing the types of patients admitted (*cream-skimming*; 4%). A second survey administered in 2007 found that more administrators (57%) believed that quality measures reflect the true quality of care, but up to 80% admitted to making no major investments in response to report cards [9].

In the United States Department of Veterans Affairs (VA), nursing homes—known as Community Living Centers (CLCs)—exhibit variable levels of measured quality [10,11]. National internal-to-VA reporting of quality measures began in fiscal year (FY) 2016 with CLC Strategic Analytics for Improvement and Learning. In early 2018, CLC Strategic Analytics for Improvement and Learning was augmented with additional data and became CLC Compare. It is modeled on Nursing Home Compare and uses CMS metrics to calculate quality performance. In addition to providing detailed information, it rates each CLC from 1 to 5 stars on overall performance using a composite measure based on unannounced surveys (ie, inspections), Minimum Data Set (MDS)–based quality measures, and staffing [12]. Until recently however, veterans and others outside of VA had no means for assessing how well CLCs perform on these important measures. In June 2018, the overall CLC Compare star ratings became accessible through the public-facing *Access to Care* website [13]. By providing simple, CLC-specific report cards, the VA aims to facilitate direct comparison with community nursing homes and provide veterans with greater information about their options for long-term care [14].

CLC Compare has substantial and immediate implications for CLCs. First, CLCs have had little time to prepare for public reporting. Through the VA Office of Geriatrics and Extended Care (GEC)’s visits to support and educate underperforming CLCs, we are beginning to understand how benchmarking affects low performers. However, we lack the means to systematically assess the impact of internal and external reporting. Second, we have anecdotal evidence that CLC staff are worried about quality comparisons between CLCs and community nursing homes. Compared with community nursing homes, CLCs fare worse on most quality measures. For example, in the first quarter of FY 2018, the VA CLC national average for the percentage of short-stay residents who reported moderate to severe pain was 33.78%; in community nursing homes, it was 13.01% [15]. In long-stay residents, the pain numbers were even worse: 32.53% in VA versus 6.62% in the community. The current CMS state-based cut point system used in CLC Compare also does not account for the system-wide national standards governing CLCs. A recent qualitative study by Miller et al [16] found that some VA staff who made nursing home referrals perceived that CLCs provide superior quality of care than other long-term care options. However, we have a limited understanding of CLC staff members’ views of more objective comparisons. Finally, CLC Compare is designed, in part, to spur improvement. However, we do not know the aspects of CLC Compare that are helpful for ongoing QI activities or in what way, if any, public reporting motivates CLCs.

Staff responses to performance metrics may not be intended. For example, in the VA, recent allegations of maladaptive responses to public reporting of VA hospital performance included selective admission of low-risk patients while turning away veterans with complicated needs [17,18]. Similar *dysfunctional* practices have been documented in community nursing homes [6,19-22]. Therefore, it is critical to understand how individual CLCs respond to CLC Compare, so that we can help (1) identify and support positive reactions and (2) ameliorate unanticipated or potentially maladaptive ones.

Having the means to accurately assess responses to report cards and the mechanisms driving QI in long-term care is the first step in building the capacity for public reporting to spur improvement [23]. The Nursing Home Compare survey, although a good first step, is not viable for current use in CLCs. It is specific to community nursing homes, whose motivations to improve quality are largely market driven [24,25] and differ from those of CLCs. Survey items are designed to capture a wide range of activities available to nursing homes in response to public reporting (eg, changes in protocols and staff allocation, work organization, and staffing). However, the survey does not examine staff members’ challenges in interpreting or acting on report card information. In addition, it does not assess the potential sources of staff member stress stemming from the public reporting process. The survey also does not assess in-depth information about 2 essential ingredients for successful QI: staff commitment to improvement and staff willingness or capacity to change day-to-day routines [8,26]. By focusing exclusively on actions that providers took in the past, the survey fails to capture information that could be leveraged for future intervention.

The GEC recently conducted a national survey to provide a snapshot of the VA’s geriatrics and extended care programs. This offers only a glimpse into CLCs’ ongoing quality assurance activities and the job types involved in quality assurance and monitoring CLC Compare data. To find critical leverage points, we need a richer survey that captures barriers and facilitators to the use of CLC Compare for QI, immediate reactions to the public reporting system, and factors that may influence its use. Our PROACTIVE (Public Reporting Responses and Opportunities Among CLC Teams: Investigating the Current Environment) study therefore proposes to adapt and expand the Nursing Home Compare survey for meaningful use by VA researchers and operations.

### Conceptual Framework: Strategic Orientation

A conceptual framework of strategic orientation guides this study because a nursing home’s principle strategic orientation is an important determinant of whether, when, and how it responds to the publication of its quality information. Major strategic orientations can be grouped into typologies. In this study, we will use the Miles and Snow typology [27] to categorize CLCs’ strategic orientation to public reporting. This valid and reliable [28] typology has been used extensively in studies of hospitals, health maintenance organizations, colleges, banks, life insurance companies, manufacturing industries [29], and community nursing homes [9]. It describes organizations as complete systems with internally consistent sets of attributes that define their dynamic interactions with the environment. In the Miles and Snow typology, there are 3 *viable* strategic types (prospector, defender, and analyzer) and 1 *nonviable* type (reactor; Textbox 1). Prospectors are innovative and growth oriented, search for new markets and new growth opportunities, and encourage risk taking. Defenders protect current markets, maintain stable growth, and serve current customers. Analyzers maintain current markets and current customer satisfaction with a moderate emphasis on innovation. Unlike the 3 viable strategies, reactors have no clear strategy, react to changes in the environment, and drift with events. In their application of the Miles and Snow typology, Zinn et al [9] found that a nursing home’s strategic type was related to the propensity to improve quality. For example, innovative and flexible nursing homes (prospectors) were more likely to leverage the *environmental shock* resulting from Nursing Home Compare to gain advantage over their competitors, for example, by investigating the causes of and acting quickly upon poor scores. Conversely, nursing homes that focused only on performing well in a limited set of core services (defenders) were more likely to take no action at all.

**Miles and Snow typology of strategic orientation.**

**Prospector**

- The nursing home responds rapidly to early signals of market opportunities to provide new services, even if some prove to be less successful than others.

**Defender**

- The nursing home focuses on providing and improving a set of services that have remained stable in time.

**Analyzer**

- The nursing home maintains a relatively stable base of services but will move into new areas that prove successful for others.

**Reactor**

- None of the above consistently describes the nursing home’s approach to providing services.

### Significance

The June 2018 launch of CLC Compare provides us with a unique and time-sensitive opportunity to gauge early CLC reactions to this significant environmental shock. Just as public reporting of VA hospital performance may have led to unanticipated negative responses among some providers [17,18], there is potential for CLC Compare to have unintended consequences on CLC staff and, ultimately, the 40,000 vulnerable veteran residents they serve. It is thus critical that we quickly and accurately understand how individual CLCs respond to CLC Compare so that we can help identify and support positive reactions among CLC staff and ameliorate unanticipated or potentially maladaptive ones.

The proposed study directly responds to the national VA priority of *greater choice for veterans*, in that it will result in a product to examine CLC staff perceptions of CLC Compare as “a readily accessible, data-rich environment to support efficient and effective health care decision making” [30]. It is also highly consistent with the VA Health Services Research and Development Service’s *aging, long-term care, and caregiving* priority domain, in which a subpriority is the “alignment of measurement with long-term services and support, home and community based services aligned with Medicare” [31].

Findings from the proposed study will immediately inform ongoing initiatives to improve CLC quality of care, thus ensuring a return on VA’s considerable investment in CLC Compare public reporting. The qualitative component of this study, in particular, is designed to elicit information not easily captured by existing surveys, that is, staff motivations to improve CLC quality, challenges with interpreting or applying information from quality report cards, staff commitment to QI, and staff willingness or capacity to change their day-to-day routines. We know that such information is essential for successfully implementing improvements in care. For example, the study findings will immediately inform VA’s CLCs’ Ongoing National Center for Enhancing Resources and Training, the platform through which GEC provides frontline QI coaching to all 134 CLCs. The GEC will also use findings to help ensure ongoing CLC staff ownership of methods for improving quality.

Veterans whose complex health needs require nursing home care represent one of the VA’s most vulnerable populations. VA’s 134 CLCs serve an average of 9991 veterans daily [32], at an annual cost of US $3.6 billion [33]. The need for skilled nursing and rehabilitative care provided by CLCs is expected to increase as the veteran population ages. Indeed, the number of highly service-connected (priority 1a) veterans for whom the VA must provide nursing home care may reach 1 million by 2023 [34]. This pilot study will help us meet our short-term goal of capturing the breadth of specific actions that CLC staff take in response to their CLC’s public data. Our immediate next step will be to widely administer the resulting survey to understand all 134 CLCs’ responses to CLC Compare, thereby furthering the scientific knowledge base on public reporting and simultaneously enabling GEC to identify and support CLCs’ positive reactions and ameliorate negative ones. Future work will examine the impact of CLC Compare on veterans’ clinical outcomes and satisfaction and inform the development and implementation of interventions to increase the use of the CLC Compare report cards for QI purposes.

VA expects veterans and their agents to be well informed about their care options and to be active in their care planning [35]. CLC Compare is one of many sources for health care information. In fact, the Access to Care website that publishes CLC Compare also includes numerous report cards on VA medical center quality (analogous to CMS’ Hospital Compare), outpatient quality, patient experience, and VA-contracted community nursing homes. Access to Care is not the only avenue through which VA and non-VA comparisons can be made. Other VA efforts to garner greater public transparency include the 2016 reintroduction of quality data from VA hospitals on the CMS’ Hospital Compare website [36]*.* We anticipate that products stemming from this pilot study will be instrumental for future studies that examine the impact of public reporting in these other critical areas.

## Methods

### Overview

Table 1 summarizes our 2 study aims, research activities, and the study participants involved in each.

**Table 1:** PROACTIVE (Public Reporting Responses and Opportunities Among Community Living Center Teams: Investigating the Current Environment) study overview.

Aim and summary	Research activities and study participants
1. Qualitative data collection and analysis	Semistructured interviews of 12 purposively selected staff in 3 CLCs^a^ (randomly selected from consistent 1-star, 3-star, and 5-star CLCs)
2. Survey adaptation, pretesting, and pilot testing	Consultation with the study advisory group, cognitive interviews of 4 staff in 1 CLC, and survey administration to the purposively selected staff at 6 CLCs (randomly selected from consistent 1-star, 3-star, and 5-star CLCs)

^a^CLC: Community Living Center.

### Study Advisory Group

This study is guided closely by a study advisory group comprising VA operations, clinical, and research leaders in long-term care quality measurement. This group participates in quarterly conference calls to provide input on study methods, provide feedback on our semistructured interview guide, and suggest improvements to the survey before pilot dissemination.

### Aim 1: Qualitative Study of CLC Staff Experiences With CLC Compare

#### Site Selection

The study sample comprises 3 CLCs (1 1-star CLC, 1 3-star CLC, and 1 5-star CLC), selected based on their star rating for the latest two quarters of CLC Compare. Specifically, we will identify all CLCs that have scored consistently in 2 consecutive reporting periods and then select 1 CLC at random from each star category (eg, a CLC with a 1-star rating in FY 18 quarter 1 and FY 18 quarter 2). We will contact the medical center directors of the selected CLCs to request study participation. If a contacted CLC declines to participate, a replacement will be selected using the procedures described above.

#### Interview Participant Recruitment

We aim to conduct 4 semistructured interviews at each CLC, for a total of 12 interviews. We will work with leadership points of contact at participating CLCs to help identify job categories involved in public reporting, that is, medical directors for geriatrics and extended care, resident assessment (ie, MDS) coordinators, quality managers, nurse managers, and assistant nurse managers, whom we will also target for participation in subsequent survey activities. We will ask the points of contact to provide VA email addresses of the identified job categories. To recruit participants, we will send email invitations. A maximum of 6 email reminders will be sent, with opt-out information. To encourage study participation, emails will include study information and letters of support from GEC, and our team has successfully used these procedures to recruit CLC staff for interviews [37]. When a staff member agrees to participate, we will set a mutually agreeable time for an interview.

#### Interview Guide Development

The content of our proposed interview guides is informed by our conceptual framework of strategic orientation [27] and studies of community nursing home administrators’ experiences with the implementation of Nursing Home Compare report cards [8,9] (refer to Multimedia Appendix 1 for the interview guide). It will be continually refined with input from the study team and our study advisory group. Specifically, we will ask participants to discuss (1) factors that motivate staff to improve CLC quality; (2) staff commitment to and difficulties with using CLC Compare for QI purposes; and (3) specific actions staff have taken in response to their CLC’s public data, including unintended consequences.

#### Interview Methods

Semistructured interviews lasting approximately 60 minutes will be performed by telephone. Interviews will be conducted by the principal investigator (CBP) and project manager (VC) and audio-recorded with the permission of study participants.

#### Data Analysis

The VA’s Centralized Transcription Services Program will transcribe all audio-recorded interviews verbatim. We will save them in NVivo 10, a qualitative coding and data management program [38], on a secure VA network. We will use an open coding approach to identify recurring patterns and themes in transcribed interviews [39], which will be deductive to the extent that it will be guided by a priori codes that we will develop before coding and inductive to the extent that new codes may be developed during coding.

Initial codes will be derived from concepts drawn from a broad literature search on nursing home public reporting and topics covered by the interview protocol. Initial codes will be refined through a process in which each researcher will read through 2 transcripts and independently generate suggestions for new codes, for modifications to or eliminations of old codes, or for combining codes into broader analytic categories. We will discuss the findings and reach a consensus on the final coding scheme. To ensure quality control, we will first code the same 2 transcripts, discuss in weekly team meetings the extent to which we consistently applied the same codes to the same text segments, and resolve discrepancies by reaching a consensus about the most appropriate code. Second, each of us will code our own set of remaining transcripts. We will continue to meet weekly to discuss and reach a consensus about new codes, insights, and challenges.

### Aim 2: Adapting a Survey to Capture CLC Staff Experiences With CLC Compare Public Reporting

#### Original Nursing Home Compare Survey

The Nursing Home Compare survey created by Mukamel et al [8] was designed to capture a wide range of activities available to nursing homes, such as changes in protocols and staff allocation, work organization, and staffing. A second iteration of the survey asked many of the same questions as the first, with additional questions seeking information on the extent to which quality measures, deficiencies, and staffing influenced medical referrals; contracts with managed care organizations; and *when* actions were taken as a direct result of the publication of the quality measures (vs *what* specific quality measures drove the actions). The original 19-item survey was estimated to take only 10 minutes to complete.

#### Adapting the Survey

We will follow standard instrument adaptation procedures to create a survey that represents CLCs’ experience with public reporting. The Nursing Home Compare survey, our conceptual framework of strategic orientation, and the public reporting literature will help define new potential domains for the survey. We will use aim 1 findings to inform mutually exclusive, overarching domains that encompass the breadth of possible CLC perceptions and reactions to public reporting (eg, *Changes to Staffing*) and then create objectives for items within each domain (eg, items within the *Changes to Staffing* domain should identify efforts to restructure existing staffing resources). After consultation with our study advisory group about the inclusion of the new domains and item objectives, we will construct survey items using language similar to the extant survey and verbatim responses from CLC staff to help reflect issues specific to the VA context. The resulting set of draft items will be shared again with the study advisory group for final approval.

We will ensure that the adapted survey, called the PROACTIVE survey, conforms to the best practices in survey design. That is, the survey will include simple wording and sentence construction to promote respondents’ accurate and reliable cognitive processing [40,41]; use *native* instead of *analytic* terms and phrases [42]; have reference periods appear as the first element in a question; have questions be explicit, specific, and of an appropriate length for the things we are asking about [43]; and incorporate definitions and instructions into the body of questions to ensure that all respondents have the same information when they answer a question.

To further ensure clarity and usability of the PROACTIVE survey, we will conduct cognitive interviews with up to 4 individuals occupying potential respondent types at 1 local VA medical center. Cognitive interviews are well-established and critical pieces of the presurvey evaluation process [44]. We will design our cognitive interviews to look specifically at participants’ experiences with comprehension of and response to questions. Following a well-established approach for performing cognitive interviews [45], we will ask respondents to independently complete the survey. A member of the study team will then review each question with the respondent to elicit information on the respondent’s interpretation of terms, the clarity of the instructions and survey items, and how the respondents answered the question. We will follow the procedures mentioned above to recruit the staff targeted by the actual survey, that is, staff involved in the public reporting process. This will include the CLC medical director, MDS coordinator, quality manager, nurse managers, and assistant nurse managers. As in aim 1, we will ask leadership to provide VA email addresses of identified CLC staff, whom we will contact separately. Each cognitive interview will last approximately 1 hour. Interviews will be audio-recorded, and respondents’ experiences with each survey item will be summarized. Potential problems with questions may include respondents’ lack of information, ambiguous terms, items not measuring intended constructs, items measuring constructs that are inapplicable to respondents, and items making discriminations that are too subtle for respondents. Problematic survey items will be revised or discarded by study team consensus.

#### Pilot Administration Site Selection

The sample will comprise 6 CLCs (2 1-star CLCs, 2 3-star CLCs, and 2 5-star CLCs), selected based on their star rating for the latest two quarters of CLC Compare. As in site selection for aim 1, we will identify all CLCs that have scored consistently over 2 consecutive reporting periods. CLCs whose staff participated in qualitative interviews (aim 1) or survey pretesting via cognitive interviews (aim 2) will be excluded from the pool of candidate sites. We will select 2 CLCs at random from each star category. The study team will recruit CLCs by emailing their leadership, explaining what is involved in study participation, and including support letters from GEC that emphasize the importance of the study to ongoing CLC QI efforts at their VA medical center. If selected CLCs do not wish to or are not able to participate, we will select a replacement from the appropriate star rating category.

#### Pilot Participant Recruitment

Although CLC frontline staff members do much of the actual QI work, they are typically not involved in making decisions about QI initiatives or where to focus resources. It is thus a more efficient use of resources in this pilot study to target staff in leadership roles that play a bigger role in their CLC’s response to quality measure data. We will recruit staff at participating CLCs involved in the public reporting process, that is, medical director for geriatrics and extended care, MDS coordinators, quality managers, nurse managers, and assistant nurse managers. We will ask site points of contact to provide VA email addresses of CLC staff in these job categories, and we will email identified staff members separately.

#### Pilot Data Collection and Management

To recruit participants, we will send email invitations. A maximum of 6 email reminders will be sent, each with opt-out information. Emails to potential respondents will include study information and letters of support from the GEC. The emails will contain a link to a web-based version of the PROACTIVE survey, administered through REDCap (Research Electronic Data Capture; Vanderbilt University) [46], which will take approximately 10 minutes to complete. Once the survey is launched at a given CLC, we will seek to maximize response rates using a data collection approach based on the method by Dillman et al [47], adapted to email and web administration. We successfully used targeted emails with links to electronic surveys in our previous studies of CLC staff, with response rates of 39%-85% [25]. We anticipate that each CLC will have a minimum of 6 staff members involved in the public reporting process (assuming an average of 2 units per CLC). On the basis of the initial administration of the Nursing Home Compare survey [8], we anticipate a minimum 50% response rate, yielding at least three completed surveys per CLC.

#### Pilot Data Analysis and Assessment of Survey’s Psychometric Properties

After the survey is closed at all 6 CLCs, data sets from RedCAP will be converted into SAS (SAS Institute Inc) data sets. Two researchers (CBP and DCM) will supervise data management and quality control. Analyses will be conducted using SAS software, version 9.4, and survey results will be examined by the quantitative study team in collaborative meaning-making sessions. The formative evaluation of our study will focus on our experiences at the level of the individual respondent and of the CLC. To assess how well our survey was received at the *individual* level, we will examine survey completion rates and completeness of data. To inform the survey’s performance at the *CLC* level, we will review notes taken at weekly meetings of the quantitative team to assess research staff time and resources required for recruiting CLCs to participate in the survey pilot and successfully launching and administering the survey at each CLC. If we collect consistent answers from survey respondents within a given CLC, a subsequent large-scale survey administration will target only 1 representative staff member at the CLC (eg, the medical director for geriatrics and extended care). If responses vary within each CLC, however, large-scale administration will mimic this pilot administration in eliciting perspectives from a wide variety of job types.

No psychometric assessments were conducted on the Nursing Home Compare survey. Therefore, we propose to establish the PROACTIVE survey’s preliminary psychometric properties by examining the distribution of responses. This will allow us to identify problematic items, that is, those with missing values or that elicit a high proportion of the most negative (floor effect) or the most positive (ceiling effect) response options. Missing items may indicate questions that are unclear or difficult to understand. We expect low rates of true missing values because truly problematic items should be eliminated during survey pretesting via cognitive interviews. Items that cluster around a single response will be flagged for possible refinement, for example, by adjusting response options to capture variations in practice that are currently being grouped into a single response category.

In analyses of preliminary survey results, responses will be aggregated and analyzed at the level of the CLC. We will calculate descriptive statistics and means for the survey items. If >50% of respondents in each CLC provide an affirmative response to an item, we will consider their CLC to have taken that specific action in response to CLC Compare.

#### Sample Size Considerations

Only a limited number of staff are involved in making decisions about QI initiatives in each CLC, and we have only 6 participating CLCs. Therefore, we do not plan to conduct robust psychometric analyses, such as exploratory factor analysis, item response theory analysis, confirmatory factor analysis, and multitrait scaling analysis, in this proposed study. Robust exploratory factor analysis and item response theory analyses require 5-15 respondents per survey item [48-51]. We estimate that the PROACTIVE survey will consist of 20 items, so we would need a derivation sample of at least 100 respondents and an equally sized validation sample. Confirmatory factor analysis requires an even greater number of respondents in a validation sample [50,52], and multitrait scaling analysis requires a sample size of at least 180 to achieve 80% power, assuming differences between correlations of moderate effect size [53]. The number of CLCs is fixed (N=134), and we will therefore sample all of them in future large-scale studies.

## Results

This pilot study was granted a human subjects research exemption from the VA Central Institutional Review Board in February 2019 and was funded in June 2019 (refer to Multimedia Appendix 2 for peer review comments). Data collection and analysis are ongoing. We expect the results of this pilot study, including qualitative findings and information about survey development, to be published in an international peer-reviewed journal in spring 2022. Preliminary results will be reported according to the consolidated criteria for reporting qualitative research [54]. Significant protocol amendments will be reported back to the research and development committee and will be reported in the primary results paper. Authorship will be granted to all participating authors according to the current principles stated by the International Committee of Medical Journal Editors.

## Discussion

### Dissemination and Implementation

The knowledge gained from our work will help our VA operational partner (GEC) refine how CLC Compare is used, ensure that staff understand and are motivated to use the data, and implement concrete actions to improve clinical quality. The dissemination of preliminary findings from this pilot study will take place in partnership with the GEC and CLC leadership and staff. GEC is very interested in (1) evidence of CLC Compare to improve CLC quality and (2) strategies for ensuring the ongoing CLC staff buy-in of continuous QI methods. The GEC has committed to using the resources of their office to promote the wider dissemination of pilot study products, and we will conduct presentations about this work to existing CLC leadership and provider groups. In addition, members of our team are actively involved in the nationwide implementation of the VA’s program to support and advance QI in CLCs (CBP and CWH) [55] and coordinate the VA’s Long-Term Services and Support Research Network (WLM). We will use these connections to further disseminate information about study results through in-person and virtual avenues. Dissemination of pilot study findings to the broader VA health care community and to the general field will be through progress reports, presentations at national conferences, and publications in peer-reviewed journals.

### Future Research

The successful completion of this pilot study will augment our limited understanding of staff reactions to the public reporting of CLCs’ quality data, their commitment to using CLC Compare, and drivers of staff involvement in CLC QI. We expect this study to (1) provide preliminary evidence of the role that CLC Compare public reporting plays in improving CLC quality and (2) result in a survey that fully captures CLCs’ experiences with CLC Compare and points to intervention opportunities. Products based on this foundational work will include larger-scale longitudinal studies to assess the psychometric properties of the PROACTIVE survey via confirmatory factor analyses; to understand all 134 CLCs’ reactions to the public reporting of CLC quality measures, including the impact of principle strategic orientation type, by using latent class analysis; to understand CLC frontline staff experiences with CLC Compare, their motivations for and challenges with QI, and adaptations to QI-related activities over time; to investigate the impact of CLC Compare on veterans’ clinical outcomes and satisfaction; and to develop an intervention project to support meaningful use by the staff of CLC Compare. It will also provide a foundation for studying the effects of public reporting in other VA clinical areas, such as overall hospital performance, the quality of outpatient care, and veterans’ experiences with health care providers [13].

## Appendix

Multimedia Appendix 1 PROACTIVE (Public Reporting Responses and Opportunities Among Community Living Center Teams: Investigating the Current Environment) interview guide.

Multimedia Appendix 2 Peer review comments from the VA Health Services Research and Development Service.

## Abbreviations

CLC: Community Living Center
CMS: Centers for Medicare and Medicaid Services
FY: fiscal year
GEC: Office of Geriatrics and Extended Care
MDS: Minimum Data Set
PROACTIVE: Public Reporting Responses and Opportunities Among Community Living Center Teams: Investigating the Current Environment
QI: quality improvement
REDCap: Research Electronic Data Capture
VA: United States Department of Veterans Affairs
