# Supplementary material for: Health Care Providers and the Public Reporting of Nursing Home Quality in the United States Department of Veterans Affairs: Protocol for a Mixed Methods Pilot Study
Source: JMIR Res Protoc. 2021 Jul 21;10(7):e23516. doi: 10.2196/23516 (PMC8339985; doi:10.2196/23516)
Supplement: Multimedia Appendix 1 [file resprot_v10i7e23516_app1.docx]

**Appendix 1.** Interview Guide

**PROACTIVE Interview**

**Introduction:**

*Thank you for agreeing to participate in this research study. Our goal is to understand CLCs’ experiences with information about CLC quality data that are reported and compared nationally. This refers to both the internal-to-VA information that includes CLC Compare and CLC SAIL, as well as the information reported on CLC Compare, including the 5-star rating, that are now on a public-facing website called Access To Care. We will call this CLC Compare data. The aim of today’s interview is to get your input on the usefulness of CLC Compare to your CLC and to learn about how your CLC has responded to CLC Compare and the 5-star ratings.*

*We’d like to remind you that your interview will be confidential and will not be shared with anyone beyond the research team. You have the right to refuse to answer any of our questions. You also have the right to request that the audio recorder be turned off, either temporarily or for the duration of the interview.*

*Do you have any questions before we begin?* **[Answer all questions before proceeding]**

**[Start recording]** We would like to confirm that you are willing to participate in this study and consent to have our conversation recorded. **[Wait for response]**

1. Please help me understand a little more about you and your facility.
   1. What is your role in monitoring, using, or reporting your CLC’s data on quality, overall or at the unit level?
   2. Please tell me a little bit about each of the neighborhoods there. What kinds of residents typically live in each?

**Questions about CLC Compare/Access to Care website**

1. Where do you think the public is getting information about CLC quality of your facility?
2. How familiar are you with the public-facing quality data from CLC Compare on the Access to Care website?
   1. Were you aware of your CLC’s scores on the internal-to-VA CLC Compare site prior to the June 2018 launch of CLC quality data on the public-facing website?

**Questions about CLC Compare/internal dashboard**

1. In general, what is your opinion of the internal CLC Compare measures and how some of that information about CLC quality is reported to the public?
2. How often do you check your CLC Compare 5-star rating and other quality measures on the internal-to-VA site? Every quarter when the scores are released? More than that? Less than that?
3. To what other CLCs do you compare your CLC Compare quality measures and survey results?
   1. How do you pick the sites to compare? For instance, do you look at the other CLCs in your VISN?
   2. To what else do you compare your CLC?

*Probe:* National CLC data? Non-VA nursing homes?

1. How does the picture painted by CLC Compare internally or externally match with how you see your CLC? How well do you think it captures what makes your CLC special/unique? Is there a difference for you between the internal picture and the one on the public facing site?

*Probe:* How well does CLC Compare reflect true strengths of your CLC? Weaknesses? [Alternative: *Do you think CLC Compare accurately reflects the quality of care provided to residents in your CLC?* In other CLCs? (i.e., those you’ve compared your CLC scores to)]

**Questions about CLC Compare in general**

1. What feedback have you received from others regarding your CLC’s star ratings or your CLC’s other quality measures?
2. From the public, residents, or residents’ family members?
3. From CLC staff or other VA personnel? (VAMC, hospital staff, etc.)

*Probes:* Media attention, response from VAMC leaders, etc.

[Did you receive guidance, resources, expectations from VA Central Office about participating in training]

1. What positive or negative consequences has your CLC experienced because of its CLC Compare scores?
2. *Probe:* Scores that are reported on the internal-to-VA dashboard
3. *Probe:* Scores that are public-facing
4. What specific actions, if any, have you taken to improve your CLC Compare scores? What part of CLC Compare did you target (QMs or survey)? How did you target it? Please be as specific as possible
   1. *Probes:* frontline watch list huddling (e.g., CONCERT activities), performance improvement projects, staff education, root cause analyses, new committees.
5. Is there anything else you’d like to add or share about your experience with CLC Compare, or anything else related to CLC quality?
