# Supplementary material for: Health Care Providers and the Public Reporting of Nursing Home Quality in the United States Department of Veterans Affairs: Protocol for a Mixed Methods Pilot Study
Source: JMIR Res Protoc. 2021 Jul 21;10(7):e23516. doi: 10.2196/23516 (PMC8339985; doi:10.2196/23516)
Supplement: Multimedia Appendix 2 [file resprot_v10i7e23516_app2.pdf]

SUMMARY STATEMENT

PROGRAM CONTACT: **(Privileged Communication)** ***Release Date:*** **09/21/2018**

Revised Date:

Application Number: **1 I21 HX002765-01**

Principal Investigator

PIMENTEL, CAMILLA BENEDICTO

Applicant Organization: EDITH NOURSE ROGERS MEMORIAL VETERANS HOSPITAL

Review Group: **HSR6**
HSR-6 Post-acute and Long-term Care

Meeting Date: **08/22/2018** ***RFA/PA:*** **HX18-002**
Council: **OCT 2018**
Requested Start: **01/01/2019**

Project Title: **Healthcare providers and public reporting of Community Living Center (CLC) quality: Investigating responses and opportunities for intervention through the PROACTIVE mixed-methods study**
SRG Action: **Impact Score:173**

Human Subjects: **20-Human subjects involved - No exemption designated**
Animal Subjects: **10-No live vertebrate animals involved for competing appl.**

Project Year	Direct Costs Requested
1	99,800
TOTAL	99,800

KEY SUMMARY POINTS:

1. The Team is well-qualified.
2. The topic of response of CLC management and operations to recent quality reporting is important and timely.
3. For a pilot the study is too complex with involvement from -3- medical centers.

DESCRIPTION (provided by applicant):

Background: In June 2018, VHA began public reporting of its 135 Community Living Centers' (CLCs') overall quality using a five-star rating system based on data from the national quality measures captured in CLC Compare. In light of the private sector's positive experience with report cards, this is a seminal moment for stimulating measurable quality improvements in CLCs. Yet public reporting of CLC Compare data raises substantial and immediate implications for CLCs. The report cards, for example, facilitate comparisons between CLCs and community nursing homes in which CLCs generally fare worse. This may lead to staff anxiety and potential unintended consequences (e.g., selective patient admissions—"cream skimming"). In addition, CLC Compare is designed to spur improvement, yet the motivating aspects of the report cards are unknown. Understanding staff attitudes and early responses is a critical first step in building the capacity for public reporting to spur quality. Specific Aims: We thus propose to adapt an existing community nursing home public reporting survey to reveal important leverage points to support CLCs' quality improvement efforts. Our work will be grounded in a conceptual framework of strategic orientation and conducted in partnership with the VA Office of Geriatrics and Extended Care (GEC). We have 2 aims. 1. Qualitatively examine a sample of CLC staff reactions to CLC Compare. 2. Adapt and expand upon an extant community nursing home survey to capture a broad range of responses, then pilot the adapted survey in CLCs. Methods: Aim 1: We will conduct interviews with staff at 3 CLCs (one 1-star, one 3-star, and one 5-star) to identify (1) specific staff actions taken in response to their CLC's public data, (2) staff commitment to/difficulties with using CLC Compare for quality improvement, and (3) factors that motivate staff to improve CLC quality. Aim 2: We will integrate these findings with our conceptual framework to adapt and expand a community nursing home survey to the current CLC environment. We will conduct cognitive interviews with staff in 1 CLC to refine survey items. We will then pilot the survey in 6 CLCs (two 1-star, two 3-star, and two 5-star) to assess survey feasibility, acceptability, and preliminary psychometric properties. Expected Results and Next Steps: We expect to develop a brief survey to be used in a future national administration to (1) identify system-wide responses to CLC Compare; (2) evaluate the impact of CLC Compare on Veterans' clinical outcomes and satisfaction; and (3) develop, test, and disseminate interventions to support meaningful use of CLC Compare for quality improvement. Knowledge gained from this pilot and from future work will help GEC refine how CLC Compare is used, ensure that CLC staff understand and are motivated to use its quality data, and implement concrete actions to improve clinical quality. Products from this pilot will also facilitate studies of the effects of public reporting in other critical VHA clinical areas. Significance & Relevance to Veterans' Health: This study responds to HSR&D's Long-Term Care priority domain and the VHA priority of Greater Choice for Veterans. Just as public reporting of VHA hospital performance led to both positive and maladaptive responses, CLC Compare may have similar impacts on CLC staff and the 40,000 vulnerable Veterans they serve. This study seizes the opportunity to identify positive, as well as unintended and potentially maladaptive, early responses to CLC Compare to help improve VHA long-term care.

CRITIQUE 1

1. Significance.

This is a new application for a pilot study attempting to study a critically important topic for the VA – the effect of quality report cards in CLC. The application is well written, timely and would provide valuable information to VHA.

2. Approach.

The investigators will utilize a mixed method approach to interview staff at 3 CLCs (a 1, 3 and 5-star facilities) to better understand the responses to public release of quality data. A second aim will use this information to expand a community nursing home survey to CLCs.

3. Innovation.

The project appears innovative in that it will produce information that will assist the VA in better using quality measurement in CLC.

4. Feasibility.

No concerns regarding feasibility.

5. Investigator Qualifications.

No concerns regarding investigator qualifications. Excellent team.

6. Resubmission Applications.

Not applicable.

7. Protection of Human Subjects from Research Risk.

No concerns regarding protection of human subjects from research risk.

8 Inclusion of Women and Minorities in Research.

No concerns regarding inclusion of women and minorities in research.

9. Budget and Period of Support.

Well described budget and period of support.

10. Sharing Research Data (Data Management and Access Plan (DMAP)).

No concerns regarding the plan to share data.

11. Overall Impression.

Excellent proposal all the way around, with strong VACO support. The topic is timely and important to VHA. The Investigative team is first rate.

12. Key Strengths.

1. Excellent team.

2. Important question.

3. Well done methods section.

13. Key Weaknesses.

1. Perhaps a bit ambitious for a pilot project.

2. The team doesn't take into consideration the potential for fluctuations in the star ratings in the sampling.

CRITIQUE 2

1. Significance.

In 2018, VHA introduced CLC quality ratings with report cards that facilitate comparisons between VA CLCs and community nursing homes. The investigators plan to collaborate with the VA Office of Geriatrics and Extended Care to evaluate and expand a brief survey (CLC Compare) to be used in future national administration to evaluate the impact of the quality ratings on CLC staff and Veterans. This study is timely. It responds to VHA Long-Term Care priorities. The project has the potential to contribute knowledge to guide quality improvement in CLCs.

2. Approach.

The research plan is well reasoned and feasible. A conceptual frame work guides the study. The approach includes interviews with staff at 1-star, 2-star and 5-star rated CLCs to identify actions taken by staff in response to CLC public data, staff commitment to/difficulties using CLC data for quality improvement and factors that motivate staff to improve CLC quality. Knowledge will be used to modify CLC Compare for use in the VA healthcare system.

3. Innovation.

The project takes advantage of a time sensitive priority- recent introduction of VHA CLC quality ratings that can be compared to community nursing homes. VHA doesn't have a survey to evaluate the impact of the report cards in which VA CLC often perform worse than community nursing homes. Understanding staff attitudes and early responses is important step in building the capacity of public reporting to improve quality. The proposed study has the potential to have a substantial impact on advancing the health and health care of Veterans.

4. Feasibility.

The project is feasible. Power calculations are not included. Instead, a convenience sample of 12 subjects from 3 randomly selected CLCs will be used. The investigators propose to qualitatively examine responses to and perceptions of CLC Compare among staff in leadership roles at CLCs with different quality ratings (1-star, 2-stars and 5-stars) using semi structured interviews and to use this information to modify the survey. The exclusion of front line staff is a significant limitation as their

cooperation is needed for successful QI efforts. The sample size could be expanded to include additional staff.

Recruitment will be by email. Estimates are for a 50% response rate yielding 3 surveys per CLC. The investigators have used targeted emails with links to electronic studies of CLC staff in previous studies with response rates of 39-85%. Alternative recruitment strategies are not proposed.

The management plan appears to be effective. The team will communicate through regularly scheduled emails and monthly meetings and the quantitative teams will meet weekly.

5. Investigator Qualifications.

The proposed study will be guided by an advisory group of VHA operations, clinical and research leaders in long-term care quality measurement. The advisory board does not include any line staff from CLCs. Representation from additional staff should be included as one of the goals of the study to study factors that motivate staff to improve CLC quality.

The PI is a Research Investigator at the Center for Healthcare Organization & Implementation Research (CHOIR) and Assistant Professor at the University of Massachusetts Medical School. She has research experience in long-term care settings. Dr. Daniel Berlowitz, is a geriatrician with extensive experience in studying VA quality of care issues. Christine Hartmann, is an established research scientist with extensive experience in VA hospital and CLC settings who has also served as PI on many projects. Consultant Susan Zickmund is an expert in qualitative research and stakeholder engagement. Her role is to guide qualitative data collection, transcription and analysis. Additional time commitment from Dr. Zickmund is recommended to insure her full participation.

6. Resubmission Applications.

This item is not applicable.

7. Protection of Human Subjects from Research Risk.

No concerns regarding protection of human subjects from research risk.

8 Inclusion of Women and Minorities in Research.

No concerns regarding the participation of women and minorities in research.

9. Budget and Period of Support.

No concerns. The PI will commit 25% FTE to this project, which should be adequate. Additional funding should be allocated for Dr. Zickmund as she is the qualitative research expert for the study.

10. Sharing Research Data (Data Management and Access Plan (DMAP)).

No concerns regarding the plan to share data.

11. Overall Impression.

In 2018, VHA introduced CLC quality ratings with report cards that facilitate comparisons between VA CLCs and community nursing homes. The investigators plan to collaborate with the VA Office of Geriatrics and Extended Care to evaluate and expand a brief survey (CLC Compare) to be used in future national administration to evaluate the impact of the quality ratings on CLC staff and Veterans.

This study is timely. It responds to VHA Long-Term Care priorities. The project has the potential to contribute knowledge to guide quality improvement in CLCs.

The research plan is well reasoned and feasible. The investigators propose to qualitatively examine responses to and perceptions of CLC Compare among staff in leadership roles at CLCs with different quality ratings (1-star, 2-stars and 5-stars) using semi structured interviews and to use this information in modifying the survey. However, the exclusion of front line staff from the interviews and from the Advisory Committee is a significant limitation as their cooperation and engagement is needed for successful QI efforts. The investigators and consultants are well qualified to conduct the study.

12. Key Strengths.

1. The project is timely and significant. In 2018, VHA introduced community living center (CLC) quality ratings with report cards that facilitate comparisons between VA CLCs and community nursing homes. The investigators plan to collaborate with the VA Office of Geriatrics and Extended Care to evaluate and expand a brief survey (CLC Compare) to be used in future national administration to evaluate the impact of the quality ratings on CLC staff and Veterans.

2. As CLCs often receive lower ratings than community nursing homes, the project has the potential to expand knowledge to guide quality improvement in CLCs and to improve Veterans' health care.

3. The research plan is well reasoned and feasible.

4. The investigators and consultants are well-qualified to conduct the study and to complete the research during the time allocated.

13. Key Weaknesses.

1. The exclusion of front line staff from the interviews and from the Advisory Committee is a significant limitation as their cooperation and engagement is needed for successful QI efforts.

2. Alternative recruitment strategies are not proposed.

3. Additional time commitment for Dr. Zickmund may be indicated as she is the expert on qualitative research methods for the study.

Administrative Note

This Summary Statement contains two final critiques only.

<u>Budget</u>

It is not clear why there are so many key personnel and sites. It is suggested that Study sites and personnel be streamlined.

MEETING ROSTER

HSR-6 Post-acute and Long-term Care
Health Services Research Parent IRG
Office of Research & Development
HSR6
08/22/2018

CHAIRPERSON(S)

DOSA, DAVID M, MD
ASSOCIATE DIRECTOR
PROVIDENCE VAMC CENTER OF INNOVATION FOR LONG TERM CARE AND SOCIAL SUPPORTS
ASSOCIATE PROFESSOR OF MEDICINE AND HEALTH SERVICE
BROWN UNIVERSITY
PROVIDENCE, RI 20015

MEMBERS

ALLEN, KELLI D., PHD *
RESEARCH HEALTH SCIENTIST
DURHAM VA MEDICAL CENTER
RESEARCH PROFESSOR
UNIVERSITY OF NORTH CAROLINA AT CHAPEL HILL
DURHAM, NC 27705

DEGENHOLTZ, HOWARD B, PHD *
ASSOCIATE PROFESSOR
UNIVERSITY OF PITTSBURGH
PITTSBURGH, PA 15261

HEBERT, PAUL L., PHD *
RESEARCH SCIENTIST
SEATTLE VA MEDICAL CENTER
RESEARCH ASSOCIATE PROFESSOR
DEPARTMENT OF HEALTH SERVICES
UNIVERSITY OF WASHINGTON SCHOOL OF PUBLIC HEALTH
SEATTLE, WA 98195

INTRATOR, ORNA K., PHD *
RESEARCH HEALTH SCIENTIST
HSR&D REAP, PROVIDENCE VA MEDICAL CENTER
PROFESSOR
UNIVERSITY OF ROCHESTER MEDICAL CENTER
ROCHESTER, NY 14642

KRESEVIC, DENISE *
CLEVELAND VA MEDICAL CENTER
ASSOCIATE DIRECTOR OF EDUCATION/ EVALUATION
UNIVERSITY HOSPITAL
CASE WESTERN RESERVE UNIVERSITY
CLEVELAND, OH 44106

MATTHEWS, JUDITH T, PHD
RESEARCH ASSOCIATE PROFESSOR
SCHOOL OF NURSING
DEPARTMENT OF HEALTH AND COMMUNITY SYSTEMS
UNIVERSITY OF PITTSBURGH
PITTSBURGH, PA 15213

MAYNARD, CHARLES, PHD *
RESEARCH HEALTH SCIENTIST SPECIALIST
SEATTLE VAMC
RESEARCH PROFESSOR
DEPARTMENT OF HEALTH SERVICES
UNIVERSITY OF WASHINGTON
SEATTLE, WA 98108

MCCARTEN, JOHN RILEY, MD *
MEDICAL DIRECTOR AND ACTING CO-DIRECTOR
MINNEAPOLIS GRECC VAMC
ASSOCIATE PROFESSOR
MEDICAL SCHOOL DEPARTMENT OF NEUROLOGY
UNIVERSITY OF MINNESOTA
MINNEAPOLIS, MN 55116

MCKEE, ANN C., MD *
NEUROPATHOLOGIST
BOSTON VA MEDICAL CENTER
PROFESSOR
DEPARTMENT OF NEUROLOGY AND PATHOLOGY
BOSTON UNIVERSITY
BEDFORD, MA 01778

PAPE, THERESA LOUISE-BENDER ,DRPH
RESEARCH HEALTH SCIENTIST
HINES VA MEDICAL CENTER
RESEARCH ASSOCIATE PROFESSOR
FEINBERG SCHOOL OF MEDICINE
NORTHWESTERN UNIVERSITY
HINES, IL 60141

SELIGER, STEPHEN L., MD, MS *
ATTENDING PHYSICIAN, MEDICAL SERVICE
BALTIMORE VAMC
ASSOCIATE PROFESSOR OF MEDICINE
DEPARTMENT OF MEDICINE
UNIVERSITY OF MARYLAND
BALTIMORE, MD 21201

SHORR, RONALD I, MD, MS *
GRECC DIRECTOR
MALCOM RANDALL VAMC
RESEARCH PROFESSOR
DEPARTMENT OF EPIDEMIOLOGY
UNIVERSITY OF FLORIDA
GAINESVILLE, FL 32608

SIMMONS, SANDRA F, PHD *
RESEARCH SCIENTIST AND DEPUTY ASSISTANT DIRECTOR
FOR RESEARCH
TENNESSEE VALLEY HEALTHCARE SYSTEM, GRECC
DEPARTMENT OF MEDICINE
INSTITUTE OF MEDICINE AND PUBLIC HEALTH
CENTER FOR QUALITY AGING
VANDERBILT UNIVERSITY, SCHOOL OF MEDICINE
NASHVILLE, TN 37232-2400

STELMACK, JOAN, OD *
CHIEF, LOW VISION OPTOMETRY
HINES VA HOSPITAL
CLINICAL ASSOCIATE PROFESSOR
ILLINOIS EYE AND EAR INFIRMARY
UNIVERSITY OF ILLINOIS
CHICAGO, IL 60612

THOMAS, KALI ST. MARIE, PHD *
RESEARCH HEALTH SCIENTIST
PROVIDENCE VA MEDICAL CENTER
ASSOCIATE PROFESSOR
DEPT OF HEALTH SERVICES, POLICY & PRACTICE
BROWN UNIVERSITY
PROVIDENCE, RI 02912

WEINER, MICHAEL, MD, MPH *
PRINCIPAL INVESTIGATOR, VA HSR&D CENTER FOR
HEALTH INFORMATION AND COMMUNICATION
RICHARD L. ROUDEBUSH VA MEDICAL CENTER
PROFESSOR OF MEDICINE
INDIANA UNIVERSITY SCHOOL OF MEDICINE
INDIANAPOLIS, IN 46202

SCIENTIFIC REVIEW OFFICER

FITZELLE, GEORGE T, PHD
DEPARTMENT OF VETERANS AFFAIRS
OFFICE OF RESEARCH AND DEVELOPMENT
HEALTH SERVICES RESEARCH AND DEVELOPMENT
WASHINGTON, DC 20420

* Temporary Member. For grant applications, temporary members may participate in the entire meeting or may review only selected applications as needed.

Consultants are required to absent themselves from the room during the review of any application if their presence would constitute or appear to constitute a conflict of interest.
